# Genome Sequence of *Campylobacter showae* UNSWCD, Isolated from a Patient with Crohn’s Disease

**DOI:** 10.1128/genomeA.00193-12

**Published:** 2013-02-14

**Authors:** Aidan P. Tay, Nadeem O. Kaakoush, Nandan P. Deshpande, Zhiliang Chen, Hazel Mitchell, Marc R. Wilkins

**Affiliations:** a Systems Biology Initiative, School of Biotechnology and Biomolecular Sciences, the University of New South Wales, Sydney, New South Wales, Australia; b School of Biotechnology and Biomolecular Sciences, the University of New South Wales, Sydney, New South Wales, Australia

## Abstract

*Campylobacter showae* UNSWCD was isolated from a patient with Crohn’s disease. Here we present a 2.1 Mb draft assembly of its genome.

## GENOME ANNOUNCEMENT

Members of the *Campylobacter* genus, including *Campylobacter jejuni* and *Campylobacter coli*, are known to play important roles in intestinal disease (1, 2). *Campylobacter showae* has been previously associated with the human oral cavity and linked with gingivitis and peridontitis (3, 4). The *C. showae* strain RM3277, isolated from the gingival crevice, has been previously sequenced and is available in the public domain as a reference. The availability of the genome sequence of a new *C. showae* strain will provide an opportunity to examine differences that may exist between oral and intestinal strains of the bacterium. The UNSWCD strain was sequenced by using an Illumina HiSeq sequencer. A total of 16,898,066 paired-end reads were generated of read length 101 bp. This constitutes a coverage equivalent to around 800×. Prior to assembly, low-quality bases were trimmed from the sequence reads by using the SolexaQA software package (5). We carried out *de novo* assembly of the reads using Velvet 1/2/06 (6) and ABySS 1.3.4 (7). Contig sequences from both assemblers were then mapped against each other using MUMmer (8). The mapped sequences were aligned to produce a consensus sequence, which formed the final set of contigs. After assembly, genome annotation was conducted using the RAST server (9). The draft genome sequence of UNSWCD was found to be comprised of 23 contig sequences with a total genome size of 2,125,173 bases and a GC content of 45.13%. In comparison, *C. showae* RM3277 consists of 33 contigs with a genome size of 2,072,007 bases and a GC content of 45.69%. The automated annotation service by RAST predicted 2,484 coding sequences (CDS) in UNSWCD, compared with 2,313 CDS in RM3277. RAST also predicted 41 RNA sequences (3 rRNA and 38 tRNA) in both the strains. Initial comparative analysis revealed that 2,158 CDS were common to the strains, while 326 CDS were found to be specific to UNSWCD and 155 specific to RM3277. One UNSWCD contig (contig 21, 41 kb in size) was suspected to be a plasmid due to its partial homology to plasmid sequences from other *Epsilonproteobacteria* and the presence of five phage-related genes. The presence of a plasmid within *C. showae* UNSWCD was verified experimentally. A cluster of genes from the CRISPR family was identified to be unique to RM3277 when compared to UNSWCD. Conversely, a set of phage proteins was found to be present in *C. showae* UNSWCD, yet absent in RM3277. Moreover, proteins belonging to a type IV secretion system were identified within the unique proteins of UNSWCD, suggesting that this strain may have acquired this system through horizontal transfer. The initial comparative genomics analysis indicated intraspecies variation and its possible relation to the bacterium’s niche within the host. However, a more detailed investigation is required to confirm this hypothesis.

### Nucleotide sequence accession numbers.

This Whole Genome Shotgun project has been deposited at DDBJ/EMBL/GenBank under the accession number AMZQ00000000. The version described in this paper is the first version, AMZQ01000000. The NCBI locus id for this submission is CSUNSWCD.
